# Structural characteristics and influencing factors of a spatial correlation network for tourism environmental efficiency in China

**DOI:** 10.1038/s41598-024-52434-4

**Published:** 2024-02-01

**Authors:** Zhenjie Liao, Lijuan Zhang, Xuanfei Wang, Shan Liang

**Affiliations:** 1 School of Management, Guangzhou Huashang College, Guangzhou, 511300 China; 2 School of Information, Guangdong University of Finance and Economics, Guangzhou, 510320 China; 3 School of Economics, Guangzhou City University of Technology, Guangzhou, 510800 China

**Keywords:** Ecology, Environmental social sciences

## Abstract

The tourism industry in China presents uneven tourism efficiency but deepening spatial associations; thus, tourism resources must be more rationally allocated. In this study, the highly efficient SBM model was used to measure the tourism environmental efficiency of 31 provinces in China. A spatial correlation network is then constructed based on the gravity model, and the structural characteristics and influencing factors of the network are analyzed. The results show that (1) the overall tourism environmental efficiency in China presents a fluctuating growth trend, with significantly higher values observed in the eastern region than in the central and western regions; moreover, the growth in efficiency in the eastern region has been relatively stable in recent years, that in the central region has increased, while that in the western region has significantly declined. (2) A spatially linked network with a stable tourism environmental efficiency structure has been formed in China. The number of network relations and density of the network fluctuate and increase, while the network efficiency continues to decrease; however, a strong small-world nature is observed. (3) An obvious network core–edge structure is observed, with Shanghai, Beijing, Zhejiang, and Jiangsu at the center showing a significant intermediary role and remote provinces such as Tibet, Xinjiang, Ningxia, and Inner Mongolia at the edge showing fewer connections. (4) The four major plates of China based on the CONCOR algorithm are sparsely connected internally and show strong inter-plate connections and spillover effects. (5) The industry support capacity difference matrix, technological development level difference matrix, transportation accessibility difference matrix, and environmental regulation level difference matrix significantly and positively affect spatial associations, while the geographical distance matrix significantly and negatively affect spatial association relationship establishment. These findings have important theoretical and practical significance for the sustainable development of tourism in China's provinces and cities.

## Introduction

In September 2020, China proposed the “dual carbon” goal at the 75th United Nations General Assembly. Over the past 3 years, various fields and industries across the country have continuously consolidated the foundation of energy conservation and emission reduction, and compacted the task of green development. The “dual carbon” goal has become a green engine leading China's high-quality development. Against the backdrop of achieving the dual carbon goal, promoting the green development of the tourism industry has become a dependent path for the high-quality development of the tourism industry in the new era. The efficiency of the tourism environment represents its level of green development, which is directly related to the coordinated development of the tourism industry and the ecological environment. Faced with the transformation of major contradictions in Chinese society, the extensive development model of the tourism industry has triggered a series of problems such as the excessive development of regional tourism resources, deterioration of the regional ecological environment, and imbalanced development of regional tourism. Strengthening regional tourism cooperation, improving tourism environmental efficiency, and promoting the improvement of tourism industry quality and efficiency have become hot topics of common concern in the current academic community. Environmental efficiency refers to the provision of products or services with competitive price advantages that meet people's pursuit of a happy life, while reducing the impact of products or services on the ecological environment and the intensity of resource consumption. In this study, to calculate the economic value of tourism economic activities and evaluate the impact of tourism economic development on the ecological environment, tourism environmental efficiency is defined as a function of the tourism economy, resources, and environment, and the ratio of investment factors, such as tourism resources, labor force, tourism capital, and environmental management, to output factors, such as tourism economic value and its impact on the environment. The higher the environmental efficiency value of tourism, the greater is the economic value of tourism and the smaller is the environmental load brought by tourism development.

Efficiency refers to the evaluation method of using resources most effectively to meet set wishes and needs under given conditions such as investment and technology^1,2^. Tourism efficiency is a comprehensive indicator that reflects the utilization level of tourism development resources and the sustainable development ability of tourism^3,4^. Improvement in tourism efficiency plays an important role in promoting the transformation of the tourism economy and the sustainable development of the tourism industry^5,6^. Tourism efficiency refers to obtaining the maximum output of tourism-related resources with minimal input within a certain time frame to meet the needs of stakeholders and maximize the total surplus^7–9^. This reflects the rationality of tourism growth^10–12^. In terms of research content, previous studies can be divided into the following types: (1) Studies that measure or evaluate tourism efficiency by constructing an index system and using data envelopment analysis (DEA)^13^ and the stochastic frontier approach (SFA)^14^. In the process of constructing the indicator system, the existing literature pays more attention to the richness of indicators. Although the number of selected indicators is large and the coverage is wide, such conditions may weaken the focus of the study and cause deviations from the core issue of tourism efficiency. (2) The spatiotemporal dynamic evolution or divergence of tourism efficiency is studied by combining the Moran index^15^, kernel density estimation^16^, and Thiel index^17^, and the types of tourism efficiency are classified. (3) Indicators are selected from the level of economic development, financial and scientific investment, education level, and geographical factors, and they are combined with econometric models to quantitatively analyze the factors influencing tourism efficiency^18,19^. The above empirical analyses are all based on “attribute data,” which can only reflect the current situation of tourism efficiency but cannot reflect the spatial correlations of tourism environmental efficiency among provinces. Moreover, the interaction mechanism among provinces is difficult to identify. Based on a literature search, we found that a number of domestic and foreign scholars have studied tourism efficiency^2,20,21^; however, relatively few studies have focused on the topic of tourism environmental efficiency^22^. The spatiotemporal differentiation of tourism environmental efficiency is influenced by multiple factors, including environmental self-purification capacity, industrial support capacity, economic development level, transportation accessibility, human capital, and technological development level, which are important influencing factors for tourism environmental efficiency. However, there are significant spatial differences and complex spatial correlations in tourism environmental efficiency among different regions in China, and factors such as economic development level and tourism industry development level further exacerbate the imbalance in spatial distribution, resulting in a trend of spatial heterogeneity in tourism environmental efficiency. Therefore, an in-depth exploration of the spatial correlation network structure of China's tourism environmental efficiency is not only a key proposition for the tourism industry to achieve sustainable development, but also a practical need to strengthen regional cooperation and build a harmonious society.

The study of spatial network structure has been a long-standing hot topic of interest for human and economic geographers^23^. Using network analysis, we can explore the spatial structure of the tourism environmental efficiency network in China and identify the inter-regional correlations. Moreover, we can analyze the influencing factors of the spatial correlation network from a relational perspective, which is more valuable than simply analyzing “attribute data” using methods such as the Moran index and spatial panel measures^24^. Scholars have constructed regional tourism association networks to investigate efficiency and conducted preliminary analyses of the overall characteristics, individual characteristics, and influencing factors of the networks^25–27^. However, few studies have explored the spatial association networks of tourism environmental efficiency. Numerous studies have shown that the network structure and relationships can have a considerable impact on the performance or efficiency of the participating subjects^28,29^. In addition, the “Matthew effect” is commonly observed in the network^30^, and regions with high tourism efficiency often occupy the central position of the network and can obtain numerous advantageous resources by virtue of their location to promote their own tourism efficiency. However, regions with lower tourism efficiency, which often occupy the edge of the network, experience fewer and less beneficial network effects. Therefore, the gap between regional tourism efficiencies may increase, which intensifies the imbalance of tourism efficiency. With increases in the degree of inter-regional linkages, the tourism efficiency of a region becomes more closely related to the tourism economy of the surrounding areas. Therefore, against the background of uneven tourism efficiency but deepening spatial associations, tourism resources must be more rationally allocated. Such improvements can be achieved by studying the spatial association network of tourism environmental efficiency in China based on the social network analysis method, exploring the tourism environmental efficiency associations among regions from a global perspective, investigating the factors that influence the spatial association network, and revealing the formation mechanism of the spatial relationship. The results presented here can provide a scientific reference for promoting the rational allocation of tourism resources and achieving balanced tourism environmental efficiency growth.

In summary, this study builds upon previous research results^30^, considered 31 provinces in China as the research object and applied the DEA method to measure the environmental efficiency of Chinese tourism from 2000 to 2020. Then, a spatial correlation network of environmental efficiency of tourism is constructed based on this method, and the overall characteristics of the network, individual characteristics, and block model are studied. Quadratic Assignment Procedure regression analysis is then introduced into the spatial correlation network. The structure of the spatial correlation network is clarified, and the characteristics, formation mechanism, and influencing factors of the spatial correlation network structure are explored. The findings have important theoretical and practical significance for the sustainable development of tourism in China's provinces and cities.

## Study design

### Tourism environmental efficiency measures

The DEA method presents certain advantages for multi-input and multi-output efficiency measures. Traditional DEA models can be divided into two categories: the CCR model based on constant scale payoffs and the BCC model based on variable scale payoffs. However, neither of these models consider the problem of slack variables for inefficiency measurements, and when multiple evaluated decision units are efficient, the problem of efficient or ineffective decision units cannot be further distinguished due to the maximum efficiency value of 1^31^. In 2002, Tone proposed the super-efficient SBM model, which effectively solves the problem of slack variables in the traditional model and the problem that the efficiency of effective decision units cannot be compared, and it ensures that decision units are temporally comparable across periods^32^. Therefore, in this paper, the output-oriented super-efficient SBM model is used to measure the efficiency of the tourism environment in 31 Chinese provinces^33^, which is calculated as follows:

1 $$\left\{ \begin{gathered} {\text{min}}_{p} = \frac{{1 + \frac{1}{m}\sum\limits_{i = 1}^{m} {\frac{{s_{i}^{ - } }}{{x_{ik} }}} }}{{1 - \frac{1}{q}\sum\limits_{r = 1}^{q} {\frac{{s_{r}^{ + } }}{{y_{rk} }}} }} \hfill \\ s.t.\sum\limits_{j = 1,j \ne k}^{n} {x_{ij} \lambda_{j} - s_{i}^{ - } \le x_{ik} } \hfill \\ \hfill \\ \sum\limits_{j = 1,j \ne k}^{n} {y_{rj} \lambda_{j} + s_{r}^{ + } \ge y_{rk} ;\lambda_{j} ,s^{ - } ,s^{ + } \ge 0} \hfill \\ \end{gathered} \right.$$

where *i* is the input index, and the value range is [1, *m*]; *r* is the output index, with the range of [1, *q*]; *ρ* indicates the efficiency value of the evaluated decision-making unit; *x*_*ij*_ represents the *i*th input data of the *j*th indicator; *y*_*rj*_ refers to the *r* output data of the *j* index; *s*^−^、*s*^+^ represent input and output relaxation variables; *λ* indicates scale benefit; *n* is the total number of indicator systems; *k* refers to the evaluated decision-making unit that has been eliminated from the *j*th index.

### Network construction and network analysis

#### Network construction

Determining association relations is the focus of studies on the spatial association network of tourism environmental efficiency in China, and most existing studies have used the gravity model to determine the spatial association relations between regions. A large number of studies have shown that spatial association relationships are limited based on geographic distance and decay^34^. The gravity model is constructed based on the principle of distance decay and the law of gravity, and the use of the gravity model to construct association networks can combine tourism environmental efficiency and economic geographic distance, which can better reveal the characteristics of spatial association^35^. A large number of scholars have used the gravity model to construct spatial association networks, and in this paper, the gravity model is modified based on the study of Liu^36^ and others, and the specific calculation formula is as follows:

2 $$F_{ij} = K_{ij} \frac{{M_{i} \cdot M_{j} }}{{[D_{ij} /(G_{i} - G_{j} )]^{b} }}$$

where *F*_*ij*_ j denotes the linkage intensity between provinces *i* and *j*; *Mi* and *Mj* denote the tourism environmental efficiency of provinces *i* and *j*, respectively; *K*_*ij*_ denotes the contribution rate of province *i* to *F*_*ij*_, *K*_*ij*_ = *M*_*i*_ /*M*_*i*_ + *M*_*j*_; *Dij* denotes the geographical distance between provinces *i* and *j*; *G*_*i*_ and *G*_*j*_ denote the economic development level of provinces *i* and *j*, respectively, and are measured by GDP per capita; and *b* is the distance decay coefficient. To study the relationship between provinces, the distance decay coefficient is usually assigned a value of 2. The linkage strength between provinces is measured by the gravity model. Subsequently, a linkage strength matrix is constructed, with the average value of each row in the matrix used as the threshold. If the linkage strength is greater than this threshold, then the value is recorded as 1, which indicates the existence of tourism environmental linkages between provinces. If the linkage strength is less than this threshold, then the value is recorded as 0, indicating the non-existence of tourism environmental linkages. Finally, a 31*31 directed binary spatial association matrix is formed.

#### Network analysis

The overall characteristics, individual characteristics, and block models of the spatially related network of tourism environment efficiency in China are studied based on social network analysis^37^. The overall characteristics are mainly divided into overall structural characteristics and small-world characteristics. The overall structural characteristics are measured by the number of network nodes and relationships, network density, network efficiency, network rank degree, and association degree, which can clarify the overall situation and overall structural characteristics of the tourism environment network. The small-world characteristics are measured by the network agglomeration coefficient and average path length, which can be used to measure the dissemination efficiency and accessibility of resources. Individual characteristics are measured by the degree centrality, intermediate centrality, and proximity centrality, which can quantify the position and power of nodes in the network and the role they play in the network. Block model analysis mainly uses the iterative correlation convergence method (CONCOR) to cluster and segment the spatially connected network, reveal the internal structure and spillover paths of the spatially connected network, and analyze the association characteristics within and between the plates, whereby the role and status of each plate in the spatially connected network can be judged.

### QAP regression analysis

This paper constructs a spatial association network of tourism environmental efficiency based on relational data. The formation of the association network is the result of multiple influencing factors, and certain correlations must occur among the influencing factors; however, such conditions cannot meet the statistical "assumption of independence of variables,” which means that the network influencing factors cannot be studied using econometric models^38^. In contrast, the Quadratic Assignment Procedure (QAP) regression analysis method, which is a social network analysis method, takes relational data as the object of study, and it does not have strict requirements for the independence of variables, and the regression results are more robust than using conventional methods^39^. QAP regression analysis is a quantitative analysis method based on matrix relationships. Compared to traditional linear regression, QAP regression can handle multicollinearity problems well, and the analysis results are also more effective and robust, making it suitable for the analysis of relational data. Therefore, in this paper, QAP regression analysis is used to study the factors influencing the spatial association network of tourism environmental efficiency to further reveal the formation mechanism of association relationships in each province.

### Data sources

Tourism environmental efficiency is a comprehensive reflection of the coordinated development of tourism and environment systems. Tourism environmental efficiency should comprehensively measure the input and output elements of tourism and the environment. Based on the principles of importance, comparability, scientificity, accessibility of the selected indicators, and previous studies, the input–output evaluation index system of tourism environmental efficiency was constructed (Table 1). Among them, the input indicators were selected from the tourism enterprises (including A-class tourist attractions, travel agencies, and star-rated hotels), the number of tourism employees, investment in tourism fixed assets, and investment in environmental pollution control. The output indicators were divided into desired and non-desired outputs. The desired output was from the total tourism revenue, while the non-desired output was the tourism carbon emission. Due to China's tourism statistics system not being sound, the current statistical yearbook data only involves the data of tourism enterprises such as A-class tourist attractions, travel agencies, and star-rated hotels. Therefore, we selected A-class tourist attractions, travel agencies, star-rated hotels, and other tourism, the number of enterprises scales, and tourism fixed asset investment as input indicators. The total investment in environmental pollution control was the input index of environmental control. Drawing on the empirical research methods of Becken et al.^40^ and Patterson et al.^41^, tourism transportation, tourism accommodation, and tourism activities are identified as the key areas of CO_2_ emissions in the tourism industry. The decomposition and aggregation method is adopted to measure the CO_2_ emissions in the tourism industry from the bottom up. The specific calculation method is as follows:

3 $$C_{{}}^{t} = \sum\limits_{j = 1}^{3} {C_{j}^{t} } = C_{1}^{t} + C_{2}^{t} + C_{3}^{t}$$

**Table 1:** Evaluation indicator system of tourism environmental efficiency.

Type	Primary indicator	Secondary indicator
Input variables	Resource input	Scale of tourism enterprises (total number of A-level tourist attractions, travel agencies and star hotels)
	Capital investment	Investment in tourism fixed assets (total investment in fixed assets of A-level tourist attractions, travel agencies and star hotels) Total investment in environmental pollution control
	Labor input	Number of tourism employees
Output variables	Expected output	Total tourism income
	Unexpected output	Tourism carbon emissions (the sum of carbon emissions from tourism transportation, tourism accommodation and tourism activities)

In the formula, *C*^*t*^ represents the total CO_2_ emissions (g) from the tourism industry in year *t*; $C_{j}^{t}$ represents the CO_2_ emissions (g) of sector *j* in year *t*; $C_{1}^{t}$ represents the CO_2_ emissions from tourism transportation in year *t* (g); $C_{2}^{t}$ represents the CO_2_ emissions (g) from tourism accommodation in year *t*; $C_{3}^{t}$ represents the CO_2_ emissions (g) from tourism activities in year *t*.

We selected 2000–2020 as the study period. The indicators' data were obtained from the China Statistical Yearbook, China Environmental Statistical Yearbook, the China Tourism Statistical Yearbook, statistical yearbooks of provinces (autonomous regions and municipalities), and the official websites of provincial cultural and tourism departments between 2001 and 2021. Missing data were obtained using the Linear interpolation method. The basic geographic data were mainly from the 1:4 million databases of the National Geographic Information Center.

## Efficiency analysis

**Figure 1:**
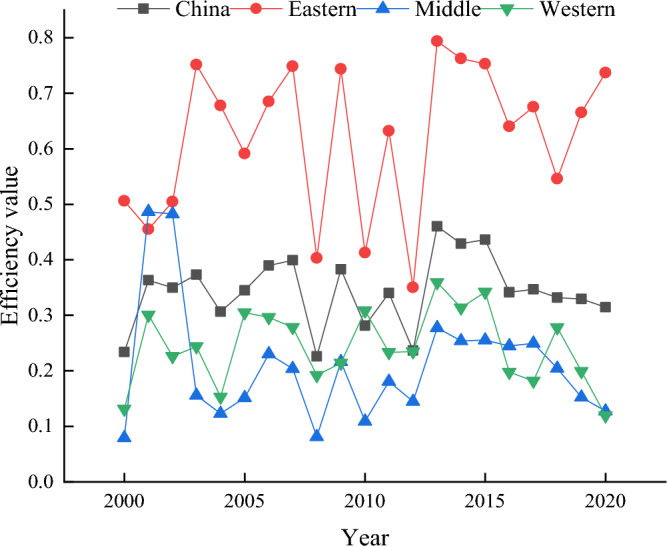
Evolutionary trend of tourism environmental efficiency in China from 2000 to 2020.

Using DEASOLVER Pro 5.0 software, the non-radial (non-oriented) variable scale payoff (VRS) super-efficient SBM model was used to measure the tourism environmental efficiency of 31 provinces and cities in China in 2000–2020, estimate the average yearly value, and adopt the usual 11:8:12 East–West regional division method to conduct a comparative analysis of different regional tourism environmental efficiencies. The mean efficiency values were compared and analyzed (Fig. 1).

**Figure 2:**
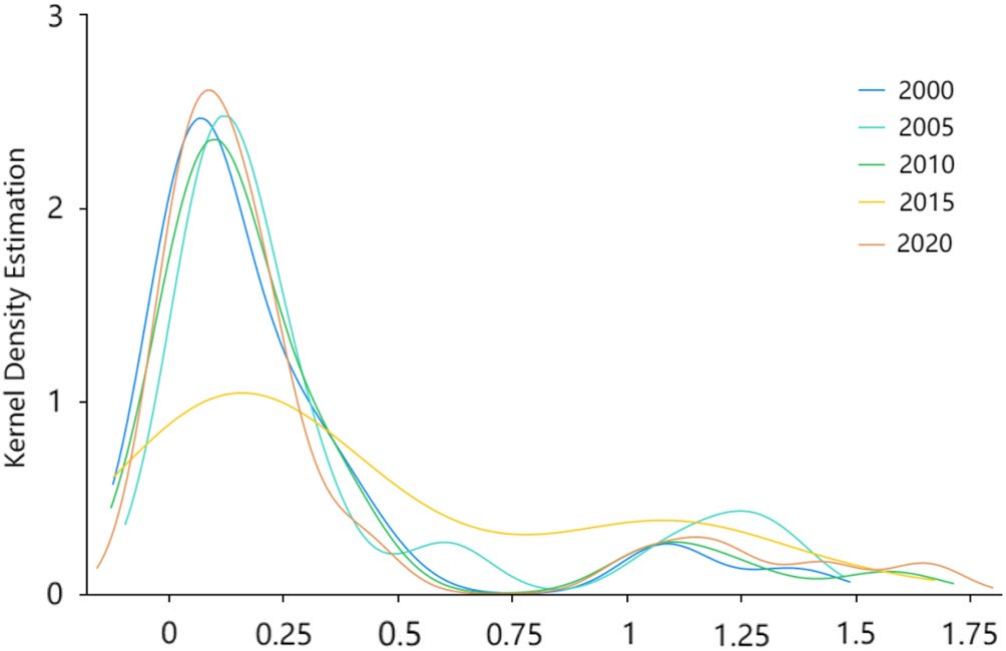
Kernel density estimation of tourism environmental efficiency in China.

The non-parametric kernel density function with normal Gaussian distribution was used to continue exploring the clustering differences in the evolution of tourism environmental efficiency over time in each province and city. Five years—2000, 2005, 2010, 2015, and 2020—were used as the observation time points for kernel density estimation^42^ to obtain the distribution at different time points (Fig. 2). The peak heights reflect the degree of agglomeration of tourism environmental efficiency in each province. China's overall tourism environmental efficiency displays a “bimodal” evolutionary distribution from left to right, with peaks from high to low (Fig. 2). This is a steady improvement trend in tourism environmental efficiency in China over time, with most provinces and cities gradually moving from low agglomeration levels to “high-low.” In 2000, the tourism environmental efficiency of most provinces and cities clustered at a low level, and after 2010, the tourism environmental efficiency of each province and city displayed varying degrees of improvement. However, there remained differences in resource endowment and tourism economy among provinces. Therefore, the gap between provinces and cities began to increase. The tourism environmental efficiency gap began to increase, forming waves of different magnitudes. However, the wave of low-level agglomeration gradually declined. In 2016, the difference in wave height of the double-peak distribution narrowed, indicating that the gap between low- and high-level tourism environmental efficiency further narrowed, gradually forming a “low–low agglomeration, high-high agglomeration.” The “bimodal club convergence” pattern was gradually formed.

## Network structure characteristics analysis

### Overall characterization

**Figure 3:**
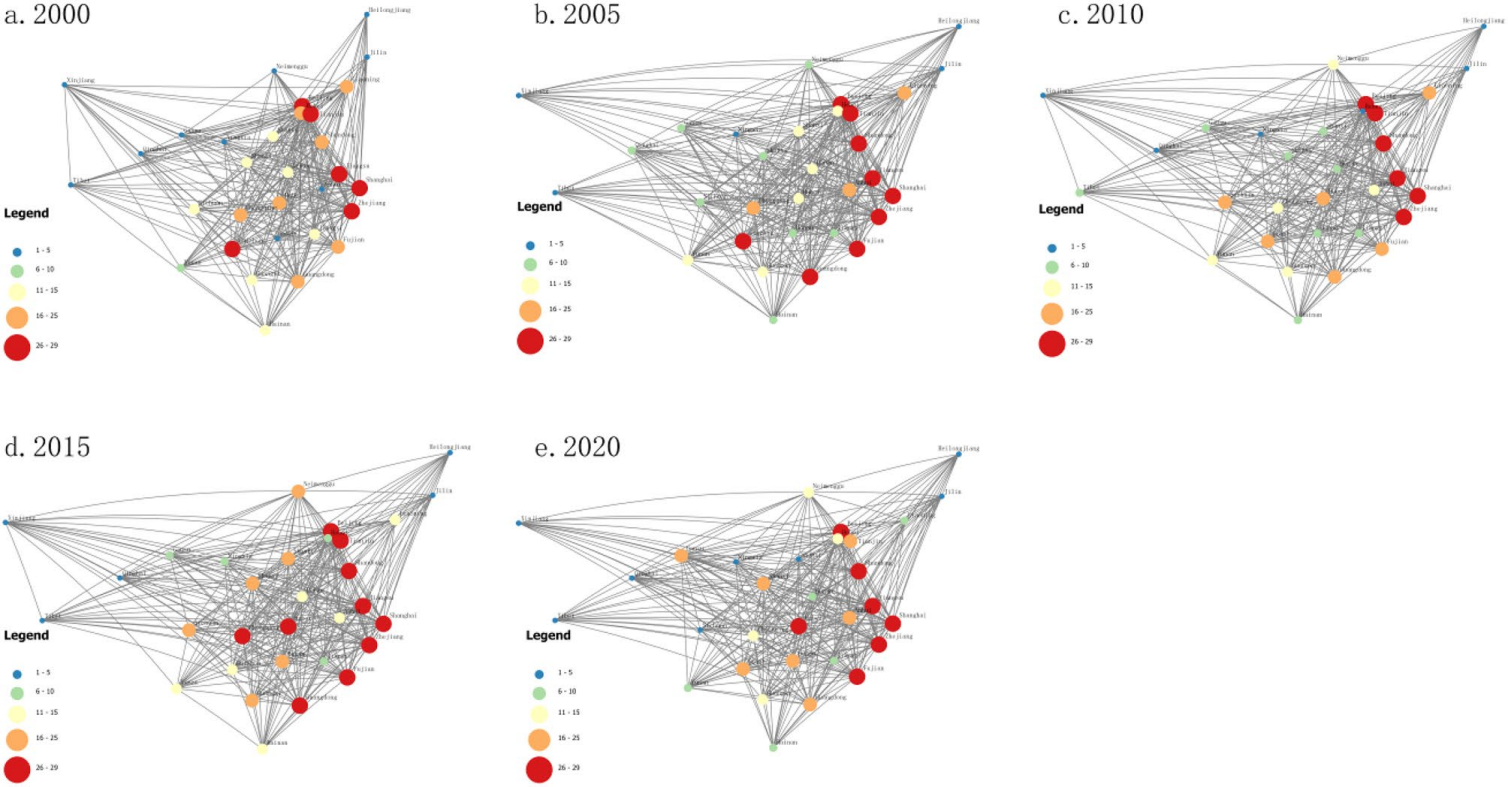
Spatial correlation network of China's tourism environmental efficiency.

Based on the spatial correlation matrix of China's tourism environmental efficiency from 2000 to 2020, a spatial correlation network is constructed. Due to space limitations, five cross-sections are selected for 2000, 2005, 2010, 2015, and 2020 in this paper and visualized using ArcGIS software, as shown in Fig. 3. The figure shows that each province's tourism environmental efficiency has an impact on neighboring or non-neighboring provinces, i.e., each province has established an association with neighboring or even distant non-neighboring provinces, thus forming an inseparable spatial association network. In 2020, for example, despite its remote location, Xinjiang had established linkages not only with neighboring provinces, such as Gansu, but also with eastern provinces and cities, such as Beijing, Tianjin, Jiangsu, Zhejiang, Shanghai, and Guangdong. These distant connections were mainly due to the promotion of the Western Development and the “One Belt, One Road” initiative, which positioned Xinjiang as the “Silk Road Economic Belt” core area. Thus, close interactions are observed between Xinjiang and other provinces.

#### Overall network structure characteristics

**Figure 4:**
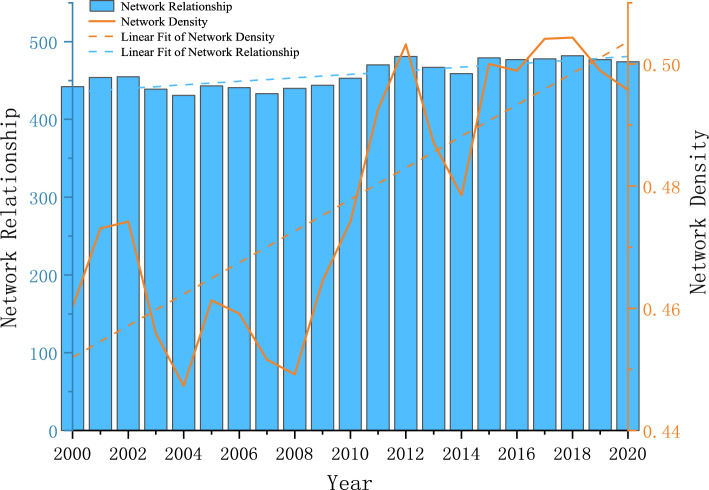
Spatial correlation network density and network relationship.

**Figure 5:**
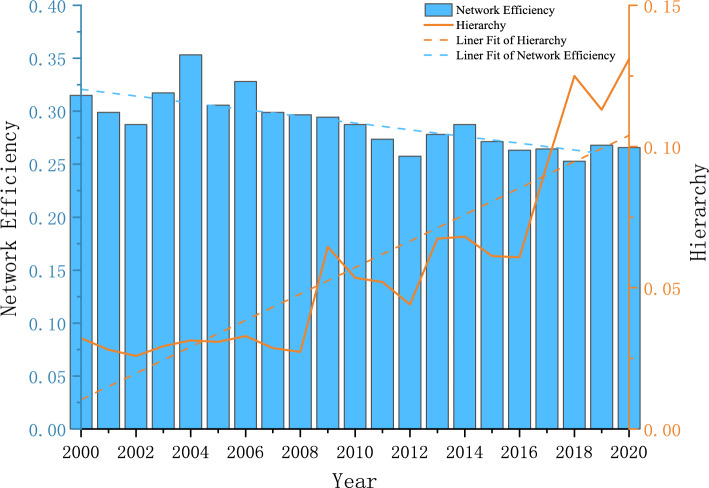
Spatial correlation network efficiency and network hierarchy.

To further characterize the overall structural features of the spatially related networks in each year, we used Ucinet software to measure the structural features of the number of relationships, network density, network relatedness, network efficiency, and network hierarchy in each year, as shown in Figs. 4 and 5. Figure 4 shows that the overall network density is small. Although according to the network relationship calculation formula n (n − 1), the maximum value is 961, which shows that the simulated number of network relationships is much higher than the real number and the tourism environmental efficiency spatial association network is still relatively loose. However, the number of network relations and overall network density present fluctuating and rising trends, and the trend line is obvious. This is mainly because the country has proposed “sustainable development,” “green low-carbon development,” “carbon peak carbon neutral,” “high-quality development” and other goals or strategies and improved the transportation infrastructure, such as high-speed rail, which has led to increased access to tourism environments in each region. The correlation degree of the spatial linkage network is 1 in all years, reflecting that, although the number of network relationships is fluctuating and developing, the network structure is more stable, and the tourism environment forms a stable spatial linkage network at the provincial scale. Figure 5 shows that the decreasing trend in network efficiency is obvious, which indicates that, with the growth in association relations, the phenomenon of multiple superposition of linkage channels between provinces gradually increases. Moreover, the number of redundant channels keeps increasing, which in turn leads to a decreasing trend in linkage efficiency. The decreasing trend in the network rank degree is obvious, which reflects the increasing association degree of tourism environmental efficiency development between provinces and the increasing trend in cross-regional synergy.

#### Small world of the web

**Figure 6:**
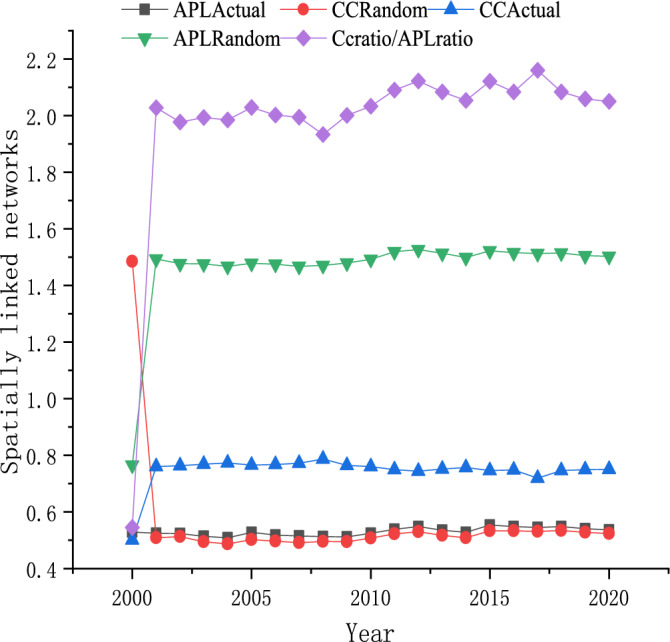
Evolution of small-world characteristics of spatial correlation network.

In social network theory, small-world features are mainly used to measure the degree of network accessibility. In this paper, we construct a random network with the same size and density as the real associated network in each year and use CCActual, APLActual, CCRandom, and APLRandom to represent the clustering coefficients and average path lengths of the real and random networks, as shown in Fig. 6. The overall clustering coefficient of the network is relatively stable, while the average path length fluctuates relatively more. However, all of these values are greater than 1, which shows that each annual spatially linked network has strong small-world characteristics^43,44^. Moreover, the network connectivity is better and regions can still use the existing network structure to achieve rapid resource flow and promote tourism environmental efficiency. However, with the gradual improvement of the network, provinces should identify redundant channels to continuously improve tourism environmental efficiency.

### Analysis of individual characteristics

The degree centrality, intermediate centrality, and near centrality of the spatial association network in 2020 were measured using Ucinet software, and the results are shown in Table 2.

**Table 2:** Individual characteristics of spatial correlation network of China's tourism environmental efficiency in 2020.

Order	Province	Degree center degree			Betweenness	Proximity to the center	
		Degree	NrmDegree	Share		inCloseness	outCloseness
1	Beijing	19	63.333	0.027	10.641	58.824	28.571
2	Tianjin	24	80	0.034	43.752	76.923	29.412
3	Hebei	19	63.333	0.027	5.08	57.692	28.846
4	Shanxi	22	73.333	0.031	9.266	68.182	29.126
5	Inner Mongolia	25	83.333	0.036	37.036	73.171	29.412
6	Liaoning	26	86.667	0.037	21.277	76.923	28.846
7	Jilin	27	90	0.039	19.677	85.714	29.412
8	Heilongjiang	20	66.667	0.029	8.53	58.824	29.126
9	Shanghai	23	76.667	0.033	13.888	68.182	29.126
10	Jiangsu	25	83.333	0.036	21.457	85.714	28.846
11	Zhejiang	21	70	0.03	5.929	55.556	29.412
12	Anhui	28	93.333	0.04	9.364	83.333	28.571
13	Fujian	15	50	0.021	0	3.226	40.541
14	Jiangxi	23	76.667	0.033	7.104	71.429	28.846
15	Shandong	28	93.333	0.04	22.141	93.75	28.571
16	Henan	21	70	0.03	7.454	61.224	29.412
17	Hubei	28	93.333	0.04	23.253	88.235	28.846
18	Hunan	26	86.667	0.037	14.445	78.947	28.846
19	Guangdong	17	56.667	0.024	3.488	54.545	28.571
20	Guangxi	25	83.333	0.036	13.29	76.923	29.126
21	Hainan	20	66.667	0.029	3.225	55.556	29.412
22	Chongqing	19	63.333	0.027	6.551	54.545	29.412
23	Sichuan	28	93.333	0.04	22.255	81.081	29.126
24	Guizhou	23	76.667	0.033	21.92	71.429	29.126
25	Yunnan	26	86.667	0.037	16.573	75	29.703
26	Tibet	15	50	0.021	0	3.226	40.541
27	Shaanxi	21	70	0.03	11.315	60	29.412
28	Gansu	23	76.667	0.033	14.82	63.83	29.126
29	Qinghai	18	60	0.026	2.89	53.571	29.412
30	Ningxia	16	53.333	0.023	1.51	53.571	29.126
31	Xinjiang	29	96.667	0.041	10.868	93.75	28.846

Degree centrality can be used to measure the position of each province in the network. Shanghai, Beijing, and Zhejiang are in the top three positions and at the center of the network, indicating that these regions are the most connected with other provinces and have the most influence on the efficiency of the tourism environment in other provinces. Tibet, Qinghai, and Ningxia, however, occupy the bottom three positions and are at the edge of the network, indicating that they are less spatially connected with other provinces and have less influence. The size of the degree centrality is related to the degree of economic development, the level of environmental regulation, and the abundance of tourism resources. Xinjiang, Hubei, and Sichuan are the centers of economic development and scientific and technological innovation in China, and their tourism industries rank among the top in the country, which greatly influences the radiation of tourism environmental efficiency to other provinces and supports their greater spatial associations. However, Inner Mongolia, Hebei, and Shanxi are relatively more backward and less associated with other provinces. Point-in degree indicates the degree of influence of a node by other nodes and reflects the beneficiary effect of the node, while point-out degree indicates the degree of influence of a node on other nodes and reflects the spillover effect of the node. Shanghai, Beijing, Zhejiang, Jiangsu, and Tianjin have high degrees of point-in, and all of them are much larger than those of point-out, which indicates that the above-mentioned regions have an obvious “siphon effect” and can absorb a large amount of advantageous resources and benefit from significant effects. The point-out degree of western regions, such as Guizhou, Chongqing, and Guangxi, is higher than the point-in degree, among which the point-in degree of Xinjiang, Ningxia, and Inner Mongolia are all 0, indicating a significant net spillover effect. The talent and resources of western regions, such as Xinjiang, Ningxia, and Inner Mongolia, flow into the central and eastern regions under the influence of the “siphon effect,” and the spillover effect is more obvious.

Overall, although the role and position of each province in the network show complex characteristics, the degree centrality and intermediate centrality indicate regions that are close to the center of the analytical results and are basically similar. However, the network core–edge structure is more significant. Shanghai, Beijing, Zhejiang, and other provinces and cities are in the center of the network and play an important intermediary role due to their significant location advantages and developed tourism economies, and they are more associated with other provinces and benefit from significant effects. Xinjiang, Ningxia, and Inner Mongolia are at the edge of the network due to their remote locations, and they are less associated with other provinces. Thus, the efficiency of their tourism environment is vulnerable to the influence of other provinces.

### Block model analysis

From the above analysis, there is heterogeneity in the status and role of each province in the network and there are significant regional differences.

To further reveal the role of each region in the network and portray the interaction between regions, China is divided into four plates based on the spatial association network of tourism environmental efficiency in 2020 using the CONCOR algorithm of the Ucinet software. The results are shown in Table 3.

**Table 3:** Division of spatial correlation network of China's tourism environmental efficiency.

Section	Section matrix				Relation-ships	Acceptance	Overflow	Expectation (%)	Actual (%)	Characte-ristics
	1	2	3	4						
1	25	12	58	72	25	156	86	40	31.42	Main overflow
2	36	8	21	28	8	42	17	20	12.98	Main overflow
3	38	7	18	26	18	42	28	20	22.43	Main overflow
4	20	6	47	52	52	3	31	10	10.87	Brokers

**Figure 7:**
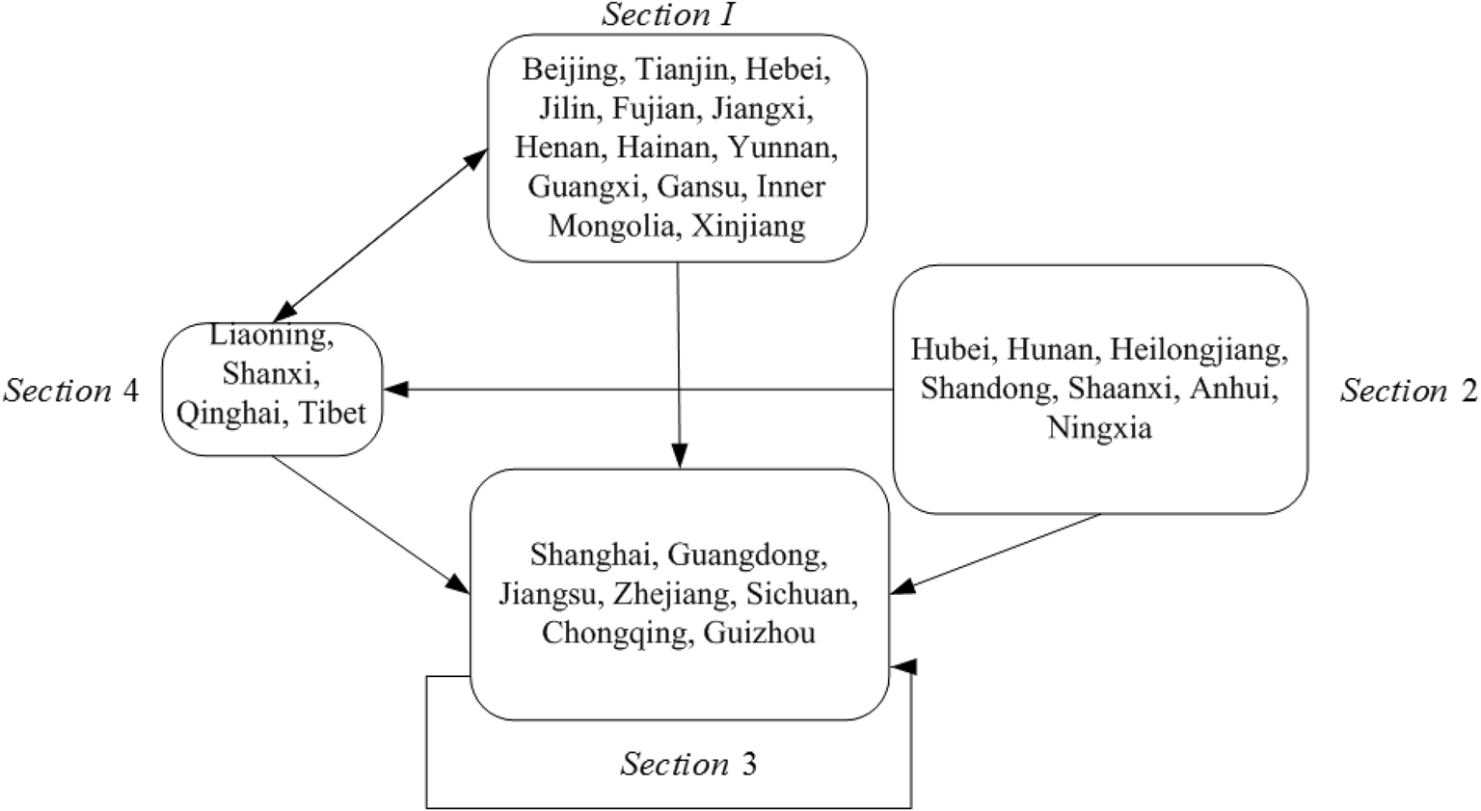
Interaction diagram of four plates.

The table shows that 346 spatial association relationships of tourism environmental efficiency in China occurred in 2020, among which 103 are intra-plate relationships and 243 are inter-plate relationships. This shows that the spatial correlations of China's tourism environmental efficiency mostly occur between plates, and the intra-plate correlations are weak. The number of intra-plate relations is 25, the number of extra-plate acceptance relations is 156, the number of spillover relations is 86, and the proportion of desired and actual internal relations is 40% and 31.42%, respectively, which shows that this plate has more external spillover relations and relatively few internal relations and can be classified as the main spillover plate^45^. The number of intra-plate II relationships is 8, the number of extra-plate receptive relationships is 42, and the number of spillover relationships is 17. The proportions of desired and actual internal relationships are 20% and 12.98%, respectively. This plate has more external spillover relationships and can be classified as the main spillover plate as well. Plate one and plate two provinces are mostly located in the central and western regions, and they present relatively backward economies and poor ecological environments but are rich in tourism resources. However, in the eastern economically developed areas, a "siphon effect" occurs under the influence of external spillover. The number of relationships within plate three is 18, the number of relationships outside the plate is 42, and the number of spillover relationships is 28. The proportion of expected and actual internal relationships is 20% and 22.43% respectively, and the relationship outside the plate is much larger than the spillover relationship; therefore, it can be defined as the main benefit plate. The number of relationships in plate IV is 52, the number of acceptance relationships outside the plate is 3, and the number of spillover relationships is 31. The proportion of expected and actual internal relationships is 10% and 10.87%, respectively. Moreover, fewer connections are observed outside the plate, and more acceptance and spillover relationships are observed, which are more balanced and assume a greater “bridge” role. Therefore, this plate can be classified as a broker plate. In the future, plate IV should strengthen its internal linkages. To show the spillover relationship between the plates, we measured the density matrix within and between plates in 2020 and measured the intra- and inter-plate-like matrix based on the overall density of the association network (Table 4). Meanwhile, to clearly show the spillover relationships among the plates, the interaction relationships among the four major plates were drawn (Fig. 7). The results show that, except for the third plate, which is more closely related internally, the other plates are more loosely related internally. In addition, the spillover effect between plates is obvious, with plates one and two mainly generating a spillover effect to plates three and four and plate four mainly generating a certain spillover effect to plates one and three. Therefore, plates one and two have obvious spillover effects and plate three benefits from the significant effect. Plate three presents a location advantage, and it has absorbed a large amount of tourism environmental efficiency growth momentum from plates one and two and continuously promotes its own efficiency. However, these characteristics are not conducive to the coordinated and balanced development of national tourism environmental efficiency.

**Table 4:** Efficiency density matrix and image matrix of China's tourism environmental efficiency.

Section	Density matrix				Image matrix			
	Section 1	Section 2	Section 3	Section 4	Section 1	Section 2	Section 3	Section 4
Section 1	0.200	0.182	0.879	0.818	0	0	1	1
Section 2	0.545	0.233	0.583	0.583	1	0	1	1
Section 3	0.576	0.194	0.467	0.542	1	0	1	1
Section 4	0.227	0.125	0.979	0.839	0	0	1	1

In general, the spatial relationships of China's tourism environmental efficiency mostly occur between plates, with relatively loose intra-plate links and relatively strong inter-plate links. The links between provinces within plates need to be enhanced. Moreover, heterogeneity occurs in the role of each plate in the network, with plates one, plate two and plate three being the main spillover plates with more external spillover relationships, and plate four being the broker plate, thus playing an important intermediary role.

## QAP regression analysis

### Selection of indicators

The previous analysis initially shows that the establishment of inter-provincial association relationships is more related to geographical location and geographical distance; therefore, we select the geographical distance between provincial capitals and consider whether the provinces are adjacent to each other as one of the influencing factors of the spatial association network. In addition, the block model analysis shows that the interaction relationship between plates is related to the level of economic development, and a greater number of plates present more developed economies and spillover relationships. Moreover, the industrial support ability, urbanization level, science and technology development level, traffic accessibility, openness to the outside world, environmental regulation level, environmental self-purification ability, relative abundance of tourism resources, human capital, and other socio-economic factors will have an impact on the tourism environmental efficiency network structure^22^. Therefore, we incorporated the above-mentioned indicators as influencing factors. As a result, the following model was constructed:

$$N_{i} = f\left( {D, \, W, \, E, \, I, \, U, \, T, \, TA, \, O, \, ER, \, ES, \, TR, \, H} \right)$$

where *Ni* denotes the spatial association network in year i; *D* represents the geographic distance matrix between provincial capital cities; *W* represents the provincial adjacency matrix, with adjacency set to 1 and non-adjacency set to 0; *E* denotes the economic development level difference matrix, measured by the GDP of each province; *I* denotes the industry support capacity difference matrix, measured by the proportion of total tourism revenue in the tertiary industry of each province; *U* denotes the urbanization level difference matrix, measured by the population urbanization rate in each province; *T* denotes the matrix of differences in the level of scientific and technological development, measured by the total investment in R&D in each province; *TA* denotes the matrix of differences in traffic accessibility, measured by the passenger turnover in each province; *O* denotes the matrix of differences in the degree of openness to the outside world, measured by the proportion of total import and export trade to GDP in each province; *ER* denotes the matrix of differences in the level of environmental regulation, measured by the proportion of total investment in environmental pollution control to GDP in each province; *ES* denotes the difference matrix of environmental self-cleaning capacity, measured by the forest cover ratio of each province; *TR* denotes the difference matrix of relative abundance of tourism resources, measured by the ratio of absolute abundance of tourism resources to the area of each region of each province; and *H* denotes human capital, measured by the number of university students in each province.

### Correlation analysis

Using Ucinet software, 5000 random permutations were selected to obtain the spatial correlation matrix of tourism environmental efficiency and the correlation coefficients of each influencing factor in 2000, 2005, 2010, 2015, and 2020, which are shown in Table 5. The coefficients of the geographic distance matrix are all negative, and the correlation coefficients of the proximity matrix are all positive, which tentatively indicates that the smaller the geographic distance between provinces, the stronger is the spatial correlation of tourism environmental efficiency. The difference in economic development level matrix is significantly positively correlated with the spatial correlation matrix, which initially indicates that the greater the difference in economic development level between regions, the more favorable is the spatial correlation relationship. The openness, government support, higher education level, and knowledge base difference matrices are all positive, which is preliminarily sign that the above indicators have a positive influence on the establishment of the tourism environment network. The industrial structure difference matrices in 2000 and 2020 have positive correlation coefficients, which preliminarily reflect that the greater the difference in industrial structure, the more favorable it is for the establishment of spatial association relationships. The correlation coefficients of the education input difference matrix and the spatial association matrix of each year did not pass the significance test, and it was preliminarily determined that the influence of education input difference on the establishment of the association relationship between provinces was not significant.

**Table 5:** Correlation analysis.

Variable	2000	Significance	2005	Significance	2010	Significance	2015	Significance	2020	Significance
*E*	0.2181***	0.04312	0.3466	0.02466	0.144**	0.04116	0.875***	0.044355	0.000729**	0.000921
*I*	0.115	0.01243	0.2822	0.04578	0.2941**	0.01202	0.89	0.033745	0.000744*	0.000734
*U*	− 0.1085	0.04304	0.4429***	0.02366	0.1838	0.04947	3.6875	0.030473	0.003073	0.000432
*T*	0.132***	0.01367	0.4739	0.01514	0.1224	0.01131	3.93***	0.029591	0.00327**	0.001006
*TA*	0.1843**	0.0343	0.4839**	0.046	0.2907***	0.01904	2.7375	0.0438***	0.002281	0.001125
*O*	0.3843	0.0426	0.1519	0.04158	0.1709	0.02948	1.347	0.020891	0.001122	0.304059
*ER*	0.2146*	0.01552	0.1783*	0.03674	0.3071**	0.03994	1.68**	0.037745	0.0014	0.358119
*ES*	− 0.3619	0.01596	0.344	0.01495	0.1718	0.01663	3.214	0.035564	0.002678	0.435545
*TR*	0.2123	0.02787	0.4785	0.03424	0.3072	0.02397	1.3975	0.028882	0.001165	0.168911
*H*	0.03	2.798	0.602**	1.613	0.38**	2.956	0.439**	0.106	0.133***	0.1379
*D*	− 0.1023*	0.0293	− 0.1704*	0.04701	− 0.3236**	0.04915	− 2.2375*	0.043791	− 0.00186**	0.196733
*W*	0.3487	0.01573	0.271	0.01024	0.3379	0.03609	3.343	0.035645	0.002786	0.167525

*p < 0.1, **p < 0.05, ***p < 0.01.

### Regression analysis

Based on the constructed model, a QAP regression analysis was conducted using Ucinet software for the spatial association matrix of China's tourism environmental efficiency with each influencing factor in 2000, 2005, 2010, 2015, and 2020. We selected 5000 random permutations, and the regression results are shown in Table 6. The adjusted *R*^*2*^ values for the 5 years were 0.1108, 0.1713, 0.3103, 0.4606, and 0.137, all of which passed the 1% significance test. This finding indicates that the changes in the above influencing factors can better explain the changes in spatial association. The regression coefficients of the geographical distance matrix in 2000, 2005, 2010, 2015, and 2020 were − 0.0426, − 0.1765, − 0.1530, − 0.0702, and − 0.4804, respectively, indicating that the smaller the geographical distance between provinces, the more conducive it is to the establishment of spatial association. The regression coefficients of the adjacent matrices are 0.0739, 0.2981, 1.2340, 0.0900, and 0.4732, respectively, the coefficients are all positive, and they all pass the significance test, indicating that the adjacent matrices are conducive to the establishment of spatial correlations between regions. The difference matrices of economic development level in 2005 and 2020 have passed the significance test at the 1% and 5% levels, respectively, and the coefficient is positive, reflecting the widening of the gap in economic development level between regions, which is conducive to the establishment of spatial correlations between regions. The difference matrices of the level of industrial support capacity in 2010 and 2015 passed the significance test at the 10% and 5% levels, respectively, and the coefficient is positive, reflecting the increase in the gap in the level of industrial support capacity between regions, which is conducive to the establishment of spatial correlations between regions. The difference matrix of urbanization level in 2000 passed the significance test at the level of 1%, and the coefficient is positive, reflecting the increase in the gap between regions in urbanization level, which is conducive to the establishment of spatial correlations between regions. The regression coefficients of the difference matrix of scientific and technological development level are 0.1102, 0.1570, 0.2201, 0.1287, and 0.1210, respectively, and the significance tests were passed at the 1%, 5%, and 1% levels in 2010, 2015, and 2020, respectively, reflecting that the difference in scientific and technological development level in the early stage has no significant impact on the establishment of spatial correlation. In recent years, the smaller the difference in scientific and technological development level, the more favorable the establishment of spatial correlation between provinces. This is mainly because the economic development of various regions, the concentration of talents and innovative resources, and the increasing differences in the level of scientific and technological development between provinces indirectly weaken the basis of inter-provincial cooperation and reduce the possibility of cooperation. The smaller the difference in the level of scientific and technological development between provinces, the more similar the cooperation needs will be, and the more conducive to the establishment of spatial relations. In 2000, 2005, 2015, and 2020, the transport accessibility difference matrix passed the significance test at the level of 5%, and the coefficient was positive, indicating that the greater the transport accessibility difference between regions, the greater the possibility of cooperation, and the more favorable the spatial correlation of tourism environmental efficiency. The difference matrix of openness degree in 2010 passed the significance test at the level of 1%, and the coefficient was positive, indicating that the greater the difference in openness degree between regions, the more conducive it was to the establishment of spatial relations. Provinces with higher openness degree interact frequently with foreign enterprises, universities, and scientific research institutions, and can receive a large number of foreign innovation resources, which is more attractive to provinces with lower openness degree in China; it is conducive to the establishment of interactive relations. In 2000 and 2005, the difference matrix of environmental regulation level passed the significance test at the 10% and 5% levels, and the coefficient was positive, indicating that the greater the difference in environmental regulation between regions, the greater is the possibility of cooperation, and the more favorable is the spatial correlation of tourism environmental efficiency. In 2000, 2010, and 2020, the difference matrix of environmental self-purification capacity passed the test at the 5%, 1%, and 10% levels, respectively, and the coefficient was positive, indicating that the greater the difference in environmental self-purification capacity between regions, the more conducive it is to the establishment of the relationship. In 2010, 2015, and 2020, the relative abundance difference matrix of tourism resources passed the test at the 10%, 5%, and 10% levels, respectively, and the coefficient was positive, indicating that the greater the relative abundance difference of tourism resources, the more conducive it is to the establishment of the relationship. In 2000, 2005, and 2010, the human capital difference matrix passed the test at the 10% level in all 3 years, and the coefficient was positive, indicating that the greater the human capital difference, the more conducive it is to the establishment of the relationship.

**Table 6:** QAP regression analysis.

Variable	2000			2010			2020		
	Coefficient	Probability1	Probability2	Coefficient	Probability1	Probability2	Coefficient	Probability1	Probability2
*E*	0.4538	0.3292	0.4388	0.3043	0.4890	0.3042	0.1455**	0.1866	0.0786
*I*	0.3607	0.3246	0.1112	0.1997*	0.4296	0.1623	0.2734	0.1813	0.2931
*U*	0.2441***	0.3606	0.0749	0.4068	0.0530	0.0526	0.4064	0.2495	0.3503
*T*	0.1102	0.2653	0.1986	0.0320***	0.2201	0.3187	0.1210***	0.1102	0.2197
*TA*	0.4181**	0.2556	0.3018	0.0868	0.4340	0.2534	0.0949**	0.0659	0.2704
*O*	0.4412	0.2594	0.2037	0.2338*	0.1413	0.1415	0.4402	0.1381	0.3473
*ER*	0.1703*	0.0873	0.4650	0.3940	0.4993	0.3530	0.4612	0.1562	0.2401
*ES*	0.0957**	0.1466	0.0512	0.4311***	0.4065	0.0415	0.1359*	0.1316	0.1378
*TR*	0.1061	0.0786	0.3822	0.3655*	0.4438	0.1149	0.0652*	0.4582	0.0622
*H*	0.2483*	0.2552	0.2838	0.4576*	0.1973	0.4144	0.1969	0.8015	0.4982
*D*	− 0.0426	0.2533	0.2389	− 0.1530	0.0650	0.0401	− 0.4804	0.7307	0.1733
*W*	0.0739**	0.6471	0.1983	1.2340*	0.0338	0.0050	0.4732*	0.3910	0.631
*R2*	0.0603***	0.2814	0.4078	0.3004***	0.1546	0.4583	0.0110***	0.3850	0.1581
*Adj R2*	0.1108***			0.3103***			0.1370***		
*Sample size*	961			961			961		

*p < 0.1, **p < 0.05, ***p < 0.01.

### Robustness tests

To further test the robustness of the regression results, thresholds were set according to different proportions of the mean values of the spatial correlation matrix of tourism environmental efficiency. Thus, 80% and 120% of the mean values were used as thresholds to construct different binary spatial correlation matrices as new explanatory variables, while the original matrix of influencing factors was applied as explanatory variables to conduct the QAP regression analysis using the above matrices.

The adjusted *R*^*2*^ value for each year passes the significance test at the 1% level, regardless of whether the 80% or 120% mean value is used as the threshold. This finding indicates that the influence factor matrix well explains the spatial correlation matrix. Meanwhile, except for the regression coefficients and significance of the industrial structure difference matrix, knowledge base difference matrix, and openness difference matrix, which are slightly different from the regression analysis results in individual years, the sign of the regression coefficients and significance of most of the other influencing factors remain unchanged, which verifies the robustness of the regression analysis results in this paper.

## Research conclusions and policy recommendations

### Research findings

A highly efficient SBM model was used to measure the tourism environmental efficiency of 31 provinces in China from 2000 to 2020, based on which a modified gravity model is used to construct a spatial correlation network of China's tourism environmental efficiency and analyze the network structure. A QAP regression analysis is then performed to study the factors that influence the spatial correlation network. We select the main factors that affect tourism environmental efficiency and the QAP regression analysis method based on existing research on the structure of tourism efficiency networks. However, due to differences in the attributes of dependent variables, there are both similarities and differences between the results of this article and the research on the structure of tourism efficiency networks. Specifically, the dependent variables used in this article are essentially matrices constructed based on the correlation structure of tourism environmental efficiency, while in previous studies on the network structure of tourism efficiency, the dependent variables were essentially matrices constructed based on the number of relationships. Therefore, the analysis results of factors affecting the tourism environmental efficiency in this article are similar to those of previous studies, further confirming the high level of economic development, the promoting effect of factors such as education level on tourism environmental efficiency, and the negative effect of transportation time and large spatial distance on tourism environmental efficiency. However, there are significant differences in the analysis of factors affecting the level of environmental regulation compared to previous studies^22^.

① China's tourism environmental efficiency has shown fluctuating growth overall, with that of the eastern region significantly higher than the national average and the central and western regions overall. The growth of tourism environmental efficiency in the eastern region has been relatively stable in recent years, that in the central region has increased, while that in the western region has shown a significant decline.
② Overall feature analysis results show that all provinces established interactive relationships with neighboring or even non-neighboring provinces, and China has formed a more stable spatial association network of tourism environmental efficiency. The number of network relationships as well as the network density show fluctuating growth, the network hierarchy and network efficiency show decreasing trends, and the redundant channels of inter-node linkages show increasing trends. However, the network still has a strong small-world nature, and each region should strengthen the identification of redundant channels to further improve the efficiency of resource dissemination.
③ By analyzing individual characteristics, we identified an obvious network core–edge structure, with provinces and cities such as Shanghai, Beijing, Zhejiang, and Jiangsu located in the center of the network and playing an important intermediary role due to their significant location advantages and more connections with other provinces, and provinces such as Tibet, Xinjiang, Ningxia, and Inner Mongolia located at the edge of the network due to their remote locations and presenting fewer connections with other provinces. Tourism environmental efficiency is vulnerable to the influence of other provinces.
④ The block model analysis showed that the spatial correlation network of China's tourism environmental efficiency forms four major segments that show sparse correlations within the segments and more close connections between segments, with strong spillover effects. Most provinces in the central and western regions having an obvious spillover effect as the main spillover plate, and the southeastern coastal region showing an obvious spillover effect and exhibiting an intermediary role as the broker plate.
⑤ QAP regression analysis results indicated that the industry support capacity difference matrix, technological development level difference matrix, transportation accessibility difference matrix, and environmental regulation level difference matrix significantly and positively affect spatial associations, while the geographical distance matrix significantly and negatively affect spatial association relationship establishment.

### Policy recommendations

Based on the above findings, the following recommendations are made to promote the overall improvement in the efficiency of China's tourism environment.

1. We recommend strengthening guidance and fully leveraging the radiation and driving roles of provinces and cities with higher tourism environmental efficiencies. Each province should comply with the trend of collaborative innovation, further promote the cross-provincial and city flow of innovative elements such as talent, technology, and capital, closely establish cooperative relationships with provinces within and between sectors, and continuously improve network density. Given the widespread popularity of the “Internet,” we recommend leveraging the role of mobile space and focusing on promoting technological innovation in production and management in the tourism field. We recommend eliminating administrative boundary constraints, avoiding the segmentation of the tourism factor market and product market, promoting the flow and transfer of resources such as technology, talent, and capital, strengthening the advantages and agglomeration effects of tourism market clusters, improving the efficiency of resource element spatial allocation, and enhancing the intensity and tightness of the spatial correlation between China's tourism environmental efficiencies. Additionally, the functions of regional tourism transportation services should be optimized, the “spatiotemporal compression” effect of transportation services should be leveraged, information technology innovation and transportation service improvement should be used as the pivot to promote the rapid flow of tourism elements in different spaces, and the spatial connection strength of tourism environmental efficiency in different provinces and cities should be strengthened via the cyclic accumulation effect.
2. Based on the current situation that tourism environmental efficiency is at a relatively low level and shows significant spatial heterogeneity, each region needs to implement policies according to local conditions and classifications, promote the transformation of the tourism industry to an intensive development mode with low energy consumption, low pollution, and low emissions, narrow regional differences, and improve tourism environmental efficiency in all aspects. Regions need to break their administrative boundaries, promote cross-regional collaboration, actively exploit the policy advantages of the “One Belt One Road” initiative, strengthen tourism collaborations with eastern and central regions, and effectively promote the spatial flow of tourism factors to "build lines, image common propaganda" and achieve regional cooperation. Moreover, a greater focus should be placed on improving the level of tourism input factors, such as resources, capital, and labor, in regions with low tourism environmental efficiency and reducing the carbon emission of tourism activities, accommodation, transportation, and other related tourism industries. In addition, the radiation-driven effect of provinces with high tourism environmental efficiencies should be strengthened in terms of the ecological development of tourism resources and configuration of tourism industry elements, and the integrated development of regional ecological tourism should be promoted.
3. Considering the identified factors that influence tourism environmental efficiency, each region can drive improvements in tourism environmental efficiency by enhancing the force of important factors. First, the regional environmental self-purification capacity should be improved. The cultivation and ecological protection of forest resources should be promoted, the emission of environmental pollutants should be reduced, and forest ecological barriers should be constructed. Second, the level of regional economic development and industrial support capacity should be vigorously improved. The transformation and upgrading of the tourism industry should be accelerated by actively adjusting and optimizing the industrial structure, downsizing tourism enterprises with low environmental performance, and comprehensively building an ecological tourism industry system. Again, the focus should be on improving the ability of science and technology innovation. Science and technological innovation should be promoted to reduce the non-expected outputs of tourism, promote the ecological economy, and encourage green-cycle and low-carbon development in the process of tourism development. Furthermore, investments in R&D funds, science- and technology-supporting facilities, and scientific research talents should be increased, and the development of technology-driven green innovation in tourism should be promoted. The development of innovative talent introduction mechanisms should also be accelerated. The government needs to increase the level of financial investment, especially for border areas, such as Tibet, Xinjiang, and Inner Mongolia, optimize the allocation of higher education resources, and focus on the cultivation of high-level tourism talents. Finally, the green construction of the tourism transportation system can be realized by exploiting the advantages of solar and wind energy and other resources to improve the level of tourism transportation cleanliness. In addition, the application of energy-saving and environmentally friendly transportation equipment in the field of passenger transportation can be improved.

## Data Availability

The datasets generated during and/or analysed during the current study are available from the corresponding author upon reasonable request.
